# Anxiety disorder and depressive disorders in teens

**DOI:** 10.1192/j.eurpsy.2024.992

**Published:** 2024-08-27

**Authors:** I. Belabbes

**Affiliations:** arazi hospital, sale, Morocco

## Abstract

**Introduction:**

Anxiety and mood disorders are frequent causes of consultation in child psychiatry. In pediatrics, they can be the cause of life-threatening or psychological complications, such as suicidal ideation, anxiety attacks, scarification or suicide attempts.

**Objectives:**

Discuss the clinical and therapeutic features of anxiety-depressive syndromes.

**Methods:**

We shed light on anxiety-depressive syndromes through the study of complex clinical cases encountered in child psychiatric hospitalization.

**Results:**

We report a case series of 10 patients, the majority of whom were female. The age range was 12 to 17 years. Clinical features included emotional manifestations such as sadness, tantrums and anxiety, as well as cognitive symptoms such as memory and concentration problems, with dark or suicidal ideation, and occasional endangerment behaviors such as scarification or suicide attempts.

Treatments range from psychosocial interventions, including therapeutic mediation, psychotherapy and social support, to pharmacological treatment with antidepressants, hypnotics, neuroleptics and, rarely, mood regulators.

**Conclusions:**

The frequency and severity of anxiety-depressive syndromes in the absence of adequate care underlines the importance of screening, early diagnosis and treatment of children with these disorders.

**Disclosure of Interest:**

None Declared

